# Transcultural adaptation and psychometric validation of the Thai-Brief Resilient Coping Scale: a cross-sectional study during the coronavirus disease 2019 pandemic in Thailand

**DOI:** 10.1038/s41598-022-26063-8

**Published:** 2022-12-13

**Authors:** Surapon Nochaiwong, Chidchanok Ruengorn, Ratanaporn Awiphan, Chabaphai Phosuya, Yongyuth Ruanta, Penkarn Kanjanarat, Nahathai Wongpakaran, Tinakon Wongpakaran, Kednapa Thavorn

**Affiliations:** 1 Department of Pharmaceutical Care, Faculty of Pharmacy, Chiang Mai University, Chiang Mai, 50200 Thailand; 2 Pharmacoepidemiology and Statistics Research Center (PESRC), Faculty of Pharmacy, Chiang Mai University, Chiang Mai, 50200 Thailand; 3 Department of Psychiatry, Faculty of Medicine, Chiang Mai University, Chiang Mai, 50200 Thailand; 4 Ottawa Hospital Research Institute, Ottawa Hospital, Ottawa, ON K1H 8L6 Canada; 5 Institute of Clinical and Evaluative Sciences, ICES uOttawa, Ottawa, ON K1Y 4E9 Canada; 6 School of Epidemiology and Public Health, Faculty of Medicine, University of Ottawa, Ottawa, ON K1G 5Z3 Canada

**Keywords:** Psychology, Health care, Medical research

## Abstract

This study aimed to examine the transcultural adaptation, construct validity, and psychometric properties of the Thai-Brief Resilient Coping Scale (BRCS) among the general population and college students through the coronavirus disease 2019 (COVID-19) pandemic in Thailand. We invited the 4004 participants to complete sets of anchor-based measurement tools, including depressive symptoms, anxiety symptoms, perceived stress, well-being, and perceived social support. The scale factor structure of the Thai-BRCS was assessed using factor analysis, and nonparametric item response theory (IRT) analysis. The psychometric properties of the Thai-BRCS for validity (convergent and discriminant) and reliability (internal consistency and reproducibility) were assessed. Based on the construct validity testing, factor analysis, and nonparametric IRT analysis reaffirmed the unidimensionality with a one-factor structure of the Thai-BRCS version. For convergent validity, the scale was significantly correlated with all sets of anchor-based measurement tools (all *P* < 0.001). The discriminant validity was satisfactory with a group of medium and low resilience and the risk of adverse mental outcomes. For scale reliability, it revealed excellent internal consistency (alpha = 0.84, omega = 0.85) and reproducibility (intraclass correlation = 0.91). The Thai-BRCS version fulfills transcultural adaptation with satisfactory psychometric properties to measure psychological resilience in the Thai population during the COVID-19 pandemic.

## Introduction

After the rapid and continuing spread of the coronavirus disease-2019 (COVID-19) worldwide, a subsequent psychosocial impact and psychological consequences have been observed^1^. Concern regarding public stress during the COVID-19 pandemic emerged due to public fear of contagion, perceived risk, widespread social media coverage, xenophobia, discrimination, and economic burden among the national and international public^2–4^. At the global public level during the COVID-19 pandemic, people are at high risk for depression (28.0%), anxiety (26.9%), posttraumatic stress symptoms (24.1%), stress (36.5%), psychological distress (50.0%), and sleep problems (27.6%)^1^. In Thailand, the burden of mental health and psychosocial problems has become a concern due to the implementation of national health and government policies that force large-scale social functions and businesses to close, resulting in job losses, facing economic burdens, and limiting individual interactions and outdoor and physical activities. As expected, the Thai population is at risk of developing mental health and psychosocial problems during the COVID-19 pandemic^1–3^.

Recently, resilience has appeared to be interested in contemporary society as an asset and fundamental to coping with stress during major disasters or public infectious events. Resilience is defined as a complex phenomenon characterized by the individual ability and capacity to overcome and recover from catastrophic events and substantial stressors^5^. During the COVID-19 pandemic, psychological resilience has been suggested as a protective factor to adapt and prevent the negative consequences of the pandemic^6^. Evidence in diverse populations showed that resilience is associated with mental health outcomes in terms of an indicator of positive adaptation to stress^7,8^. A systematic review of 60 studies, including children, adolescents, and adults, shows that trait resilience is negatively correlated with depression and anxiety, whereas positively correlated to life satisfaction and positive affect^7^. The literature among older adults in Thailand suggested that factors related to resilience are physical decline, chronic illness, psychological responses such as fear, anxiety, stress, loneliness, sense of control, emotional management, religion, and wisdom^9^. Moreover, resilience has been identified as a potential predictor of quality of life among older adults in Thailand^10^. In recent years, resilience is also an interest in the younger population due to the rising in mental health cases. However, existing tools for resilience assessment in Thailand are focused on some populations rather than the general population, such as the Connor-Davidson Resilience Scale for younger or the Thai Elderly Resilience Scale for older people^11,12^.

To date, the original English-Brief Resilient Coping Scale (BRCS) version has been widely used, translated, and validated to measure the degree of psychological resilience in several populations, both mental illness and nonmental illness in different countries, as it is simple to apply in community practice^13–15^. This scale has been established with good psychometric properties in terms of validity and reliability in several languages^16–18^. Unfortunately, the original-BRCS version has not been translated and established cross-cultural adaptation among Thai-speaking populations. Moreover, the comprehensive psychometric properties of BRCS have not been evaluated, particularly, during public health events in Thailand. For practicability, the Thai-BRCS version with four items is easy to apply and has potential value to recognize specifically psychological resilience during a crisis in Thailand. As validation of the Thai-BRCS performance will facilitate other countries to address that this tool not only helps to measure coping with stress but also reflects the success of target intervention in assessing responses to public mental health.

A valid and standardized measurement tool is needed during public mental health responses to help screen and target the population and track public psychological resilience. As such, this study aimed to examine the transcultural adaptation, construct validity, and psychometric properties of a measure of resilient coping, the Thai-BRCS version among the general population and college students through the COVID-19 pandemic in Thailand. As highlighted in psychosocial consequences due to COVID-19, we hypothesized that the Thai-BRSC 4-item contains good properties of resilience measure and correlated with adverse mental health outcomes.

## Methods

### Study design and study population

Based on the Health Outcomes and Mental Health Care Evaluation Survey: Under the pandemic situation of COVID-19 (HOME-COVID-19)—a nationwide nonprobability repeated Internet survey, this study is a part of survey wave I (April 21 to May 4, 2020) during the first wave of the COVID-19 pandemic and lockdown in Thailand (details of the study are described elsewhere)^4,19^. The study was approved by the Institutional Review Board of the Faculty of Public Health (ET010/2020) and the Faculty of Pharmacy (23/2563) of Chiang Mai University. This study was reported in accordance with the Strengthening Reporting of Observational Studies in Epidemiology Statement^20^ and Improving the Quality of Web Surveys: The Checklist for Reporting Results of Internet E-Surveys^21^.

In brief, the survey questionnaires were distributed using the SurveyMonkey^®^ platform through various social media networks, including public websites, Facebook, LINE, Twitter, and Instagram. All respondents provided their written informed consent, which was included in the screening questionnaire before participating in this survey to adhere to the physical distancing strategy protocol during lockdown in Thailand. Potential participants were invited to complete a set of questionnaires, including participant characteristics and specific tools regarding mental health issues and psychosocial problems.

Eligible participants in this study included: (i) Thai citizens, permanent residents, or non-residents with work permits aged ≥ 18 years on the date of the survey; (ii) could read and communicate in the Thai language; and (iii) could access the Internet during lockdown and provide their online consent. However, incomplete respondents or participants who spent less than 2 min or more than 60 min on the survey were excluded from this study.

### Translation and adaptation of Thai-BRCS

The original English-BRCS version is a five-point Likert scale consisting of four items that capture tendencies to cope with stress in a highly adaptive manner and an easy to complete the questionnaire. The total score ranges from 4 to 20, with a higher score indicating greater resilience to cope. The validity and reliability of BRCS have been established on a satisfactory scale in individuals with rheumatoid arthritis. The internal consistency and test–retest reliability (stability) with Cronbach’s α reliability and intraclass correlation were 0.69 and 0.71, respectively. For scale interpretation, a BRCS score of ≤ 13 points, 14–16 points, and ≥ 17 points were classified as low-, medium-, and high resilience copers, respectively^13^.

In accordance with the standard protocol, the original-BRCS version was translated by a professional lay English-language translator to establish public utility. To ensure readability and transcultural adaptation, the Thai-BRCS draft was then reviewed and refined by two independent professional translators, two psychiatrists, two methodologists, and one social scientist. The back-translation process was performed as followed: the preliminary Thai-BRCS version was then re-translated by another professional translator to English language and reconciled with the original-BRCS version. Any discrepancies were reached by a final consensus through discussions by the investigators team. Finally, the pilot Thai-BRCS was given to a group of 30 general population to verify its reliability and scale utility. The pilot test of Thai-BRCS indicated an acceptable internal consistency (Cronbach’s α, 0.80)^19^.

### Statistical analysis

Sample size estimation was calculated based on two parameters, including a stable structure for factor analysis based on the rule of thumb, which is 10–15 cases per question, and statistically significant for convergent validity with a fair correlation statistic (> 0.20). A minimum target of 259 per subpopulation of the general population and college students, with a total of at least 518 respondents were needed to ensure a 0.05 type I error and power of 90%.

All analyses were performed using Stata 14.0 (StataCorp, LP). The two-tailed tests were conducted with a type I error rate of 0.05. Descriptive statistics were used and expressed as frequency and percentage, mean ± standard deviation (SD), or median with a range (min–max) as appropriate. An independent *t* test or Wilcoxon rank-sum test was used for continuous data and Fisher’s exact test for categorical data to examine the differences in baseline characteristics between subpopulation groups (general population vs. college students). Regarding the Thai-BRCS, the item scores were summarized descriptively with the normality of the score distribution assessed by the floor/ceiling effect, skewness, and kurtosis tests. To address the level of significance, the confidence intervals (CIs) of the correlation statistics were estimated using the bootstrap resampling approach. All analyses were performed according to the whole sample population and separately based on the general population and college students. The overall psychometric properties of the Thai-BRCS version were based on the validity and reliability of the scale performance as follows:

#### Construct validity

Eligible participants were divided into a 1:1 ratio using exploratory and confirmatory factor analyses to test for the structure of the scale. The dimensionality of the Thai-BRCS version was investigated using exploratory factor analysis with a principal factor extraction method. Prior communalities were assessed and the factor was orthogonally rotated using the varimax criterion. The Kaiser–Meyer–Olkin measure and Bartlett test of sphericity were performed to ensure the appropriate use of factor analysis. Kaiser–Meyer–Olkin value of > 0.8 and Bartlett test with a *P*-value of < 0.05 are recommended for sampling adequacy and the suitability of the data for factor analysis, respectively. We used Eigenvalues with a criterion of > 1.0, along with the scree plot to establish the number of factors retained^22,23^. A parallel analysis was performed to confirm the optimal threshold for the number of factors retained.

Next, a confirmatory factor analysis with a maximum likelihood estimation was performed to reaffirm the factor structure based on the exploratory factor, as described previously. To determine the suitability of the tested model, specific fit indices, including the root mean square error of approximation (RMSEA, < 0.100), standardized root mean squared residual (SRMR, < 0.100), comparative-fit index (CFI, > 0.900), and non-normed fit index/Tucker-Lewis Index (TLI, > 0.900) were used^4,24^. Moreover, the coefficient of determination (R-squared) and item-scale correlation (standardized factor loading) should be at least 0.30 and 0.40, respectively, to establish acceptance of the final structure of the Thai-BRCS version. In addition, we performed the hierarchy of measurement invariance approach based on sample population (general population or college students), sexual identity (male, female, or others), and religion (irreligion, Buddhist, or Christian/Muslim/others). We followed this approach in order by (i) configural invariance, (ii) metric or weak invariance, (iii) scalar or strong invariance, (iv) strict or residuals invariance, and (v) strict invariance plus factor means (Appendix, eMethods)^25–28^. The constraints are cumulative and compared through the hierarchy^29^.

Finally, a nonparametric item response theory analysis was performed to confirm the unidimensional set of the Thai-BRCS version concerning the relationship between the latent trait and the responses to the items. The item response theory analysis was assessed based on fundamental assumptions, including unidimensionality, local independence, and monotonicity. Based on the traces of the items, the Loevinger’s H coefficients (*H*^*s*^) < 0.3, 0.3–0.4, and > 0.4 denote as poor, medium, and strong scalability properties, respectively. To determine the fundamentals of the nonparametric item response theory assumption described above, the monotonicity assumption criterion should be less than 80^4,30^. The item characteristics curve analysis was used to assess the characteristics of individual items within the scale, and to investigate the correlation between the predicted participants; response to an individual item and the underlying construct (expectations). Furthermore, the differential item functioning (DIF) was also assessed according to sample population (general population vs. college students), sexual identity, and religion to explore the impact of participant characteristics on individual items, with a *P*-value < 0.05 indicated as having significant DIF.

#### Convergent validity

Theoretically, convergent validity describes the degree to which the proposed assessment converges with other relevant measures. Bivariate analysis using Pearson’s or Spearman’s correlation coefficients and 95% CI between the Thai-BRCS version and the proposed tools on mental health and psychosocial problems during the COVID-19 pandemic. Correlation statistics were classified as low (< 0.3), moderate (0.3–0.5), and high (> 0.5)^31^. Mental health issues and psychosocial tools for the convergent validity test in this study included the following:

(i) Depressive symptoms: Patient Health Questionnaire-9 (PHQ-9) comprises nine questions to quantify the severity of depressive symptoms. A higher score indicated greater severity of the depressive. An optimal cut-off score of ≥ 10 points was used to identify the general population with depressive symptoms^32,33^. PHQ-9 revealed satisfactory reliability, with a Cronbach’s α of 0.79^34^.
(ii) Anxiety symptoms: Generalized Anxiety Disorder-7 (GAD-7) with seven questions was used to measure anxiety symptoms. A higher score indicates greater severity of anxiety. A cut-off score of ≥ 8 points was used to identify anxiety symptoms^35^. This scale had excellent psychometric properties, with a Cronbach’s α of 0.92^36^.
(iii) Perceived stress: Perceived Stress Scale-10 (PSS-10) consists of 10 questions with a higher score indicating a higher degree of stress. A cut-off point of ≥ 14 was used to indicate perceived stress. PSS-10 revealed acceptable psychometric properties, with a Cronbach’s α of 0.85^37^.
(iv) Well-being: The World Health Organization-Five Well-Being Index (WHO-5) with five questions was used to measure health-related personal well-being, with a higher score indicating a high well-being index. A cut-off point of < 50 points was considered a low well-being index. WHO-5 showed good psychometric properties, with a Cronbach’s α of 0.87^38^.
(v) Perceived social support: Multidimensional Scale of Perceived Social Support (MSPSS-12) consists of 12 questions to measure individual perceptions of external social support. A cut-off score of < 36 points was considered an individual with low perceived social support. This scale had excellent internal consistency, with a Cronbach’s α of 0.92^39^.

Furthermore, multivariable linear regression was also performed to confirm the linearity of the association between the Thai-BRCS version summary score and a priori tools for convergent validity testing.

#### Discriminant validity

Based on the interpretation of the original-BRCS version, discriminant validity was evaluated by a known group comparison between the high resilient copers group (BRCS score of ≥ 17 points) and medium resilient copers group (14–16 points), and low resilient copers group (≤ 13 points) using multivariable logistic regression. Compared to the highly resilient copers group, we expected that participants with medium or low resilience would experience adverse mental health and psychosocial problems in terms of depressive symptoms, anxiety, perceived stress, low well-being, and low perceived social support.

In addition, the area under the receiver operating characteristic curve (AuROC) statistic was assessed based on the degree of resilience during the COVID-19 pandemic (low, medium, and high resilience copers) and the risk of adverse mental health and psychosocial problems. The AuROC curves above the null line with a value of 0.5 are considered to have a reasonable discriminating ability to distinguish participants with and without adverse mental health and psychosocial problems^40,41^.

#### Reliability

To determine the internal consistency reliability and the degree to which every item on a scale measures the same construct, Cronbach’s α and McDonald’s ω coefficient were estimated with 95% CIs. The values of at least 0.70 indicated an acceptable reliability of the scale. Furthermore, the item-total correlations between 0.20 and 0.80 were considered acceptable^42^.

Moreover, the intraclass correlation coefficients based on the convenient sub-cohort (n = 409) between the first and second entries on day 3–5 were estimated to establish test–retest reliability (scale stability). The intraclass correlation coefficients of 0.7–0.8 and ≥ 0.8 denote substantial and almost perfect scale stability, respectively^4,42^.

## Results

### Baseline characteristics

Based on data from the first wave of HOME-COVID-19, 4004 participants who completed the set of measurement tools of interest were considered in this study, including 2415 general population and 1589 college students. Overall, most of the participants included were female (65.4%), with a mean (SD) and median (range) age of 29.1 (10.8) and 25 (18–79) years, respectively. As expected, a substantial difference in participant characteristics was observed among the subpopulation. Of these, individuals among college students were younger, single, and irreligious compared to the general population. However, according to the summary BRCS score, there was no statistical significance among the subpopulation, with a mean (SD) of 13.9 (3.1). Details of the whole sample characteristics and subpopulation cohort, including the general population and college students are described in Table 1. Based on a ratio of 1:1, 2002 (1207 general population and 795 college students) and 2002 (1208 general population and 794 college students) were randomly divided to test for exploratory and confirmatory factor analyses, respectively.

**Table 1:** Participant characteristics of the study population.

Variable	Overall (n = 4004)	General population (n = 2415)	College students (n = 1589)	*P* value
**Sociodemographic**				
Age, year, mean (SD); median (range)	29.1 (10.8); 25 (18–79)	34.6 (10.7); 32 (18–79)	20.7 (2.2); 21 (18–47)	< 0.001
Sexual identity				
Male	1231 (30.7)	887 (36.7)	344 (21.7)	< 0.001
Female	2619 (65.4)	1457 (60.3)	1162 (73.1)	
Others	154 (3.9)	71 (3.0)	83 (5.2)	
Marital status				
Single	3208 (80.1)	1626 (67.3)	1582 (99.6)	< 0.001
Married/domestic partnership	693 (17.3)	687 (28.5)	6 (0.4)	
Divorced/widowed/ separated	103 (2.6)	102 (4.2)	1 (0.1)	
Religion				
Irreligion	375 (9.4)	186 (7.7)	189 (11.9)	< 0.001
Buddhist	3454 (86.3)	2128 (88.1)	1326 (83.4)	
Christian/Muslim/others	175 (4.4)	101 (4.2)	74 (4.7)	
Living status				
With family	3164 (79.0)	1833 (75.9)	1331 (83.7)	< 0.001
With others	264 (6.6)	174 (7.2)	90 (5.7)	
Alone	576 (14.4)	408 (16.9)	168 (10.6)	
Household family member				
≤ 4	3102 (77.5)	1937 (80.2)	1165 (73.3)	< 0.001
> 4	902 (22.5)	478 (19.8)	424 (26.7)	
History of mental illness				
No	3645 (91.0)	2220 (91.9)	1425 (89.7)	0.017
Yes	359 (9.0)	195 (8.1)	164 (10.3)	
Quarantine status				
Never	1781 (44.5)	1381 (57.2)	400 (25.2)	< 0.001
Past	1575 (39.3)	765 (31.7)	810 (51.0)	
Current	648 (16.2)	269 (11.1)	379 (23.8)	
Willingness to quarantine				
Yes	3241 (80.9)	2007 (83.1)	1234 (77.7)	< 0.001
Not sure	638 (15.9)	352 (14.6)	286 (18.0)	
No	125 (3.1)	56 (2.3)	69 (4.3)	
**Mental health and psychosocial score, mean (SD); median (range)**				
Resilient coping—BRCS	13.9 (3.1); 14 (4–20)	13.9 (3.2); 14 (4–20)	13.9 (2.8); 14 (4–20)	0.952
Depression—PHQ-9	8.1 (6.1)	7.3 (5.9); 6 (0–27)	9.3 (6.2); 8 (0–27)	< 0.001
Anxiety—GAD-7	4.7 (4.7); 4 (0–21)	4.4 (4.6); 3 (0–21)	5.3 (4.8); 4 (0–21)	< 0.001
Perceived stress—PSS-10	17.4 (6.7); 17 (0–40)	16.8 (6.6); 17 (0–40)	18.3 (6.8); 18 (0–40)	< 0.001
Well-being index—WHO-5	52.2 (21.6); 56 (0–100)	53.7 (21.8); 56 (0–100)	49.9 (21.1); 52 (0–100)	< 0.001
Perceived social support—MSPSS-12	59.1 (13.7); 60 (12–84)	58.8 (14.1); 60 (12–84)	59.5 (13.1); 60 (12–84)	0.101

Data are expressed as the number (percentage) of participants, unless otherwise indicated.

*BRCS* Brief Resilient Coping Scale—4-items, *GAD-7* Generalized Anxiety Disorder—7-items, *MSPSS-12* Multidimensional Scale of Perceived Social Support—12-items, *PHQ-9* Patient Health Questionnaire—9-items, *PSS-10* Perceived Stress Scale—10-items, *SD* standard deviation, *WHO-5* World Health Organization Five Well-Being Index—5-items.

### Dimensionality and construct validity

According to the item analysis, no items with floor or ceiling effects were identified (Table 2). The parallel analysis and scree plot illustrated a one-component factor for the Thai-BRCS version with four items. The exploratory factor analysis (n = 2002) with a principal factor extraction method demonstrated that individual items had factor loadings of > 0.71 across the different subpopulations (Appendix, Table S1). Based on the overall population, the single component extracted for the exploratory factor analysis described 68.0% of the variance in the Thai-BRCS version (71.0% for the general population and 63.0% for college students, Appendix, Table S1).

**Table 2:** The Thai-BRCS version questionnaire and standardized factor loading (n = 2002).

Item (in Thai)	Scoring structure	Mean (SD); median (range)	Ceiling effect (%)	Floor effect (%)	Skewness	Kurtosis	Standardized factor loadings (95% CI)^†^	R^2^
Item 1: I look for creative ways to alter difficult situations (ฉันมองหาวิธีการใหม่ ๆ ที่ดี ในการจัดการกับสถานการณ์ที่ยากลำบาก)	5-point Likert scale: 1–2-3–4-5	3.4 (0.9); 3 (1–5)	2.7	10.2	− 0.20	2.99	0.72 (0.70–0.73)	0.51
Item 2: Regardless of what happens to me, I believe I can control my reaction to it (ไม่ว่าจะเกิดอะไรขึ้นกับฉัน ฉันเชื่อว่าฉันสามารถควบคุมการกระทำของฉันได้)	5-point Likert scale: 1–2-3–4-5	3.5 (0.9); 4 (1–5)	2.3	12.4	− 0.35	2.99	0.82 (0.80–0.83)	0.67
Item 3: I believe that I can grow in positive ways by dealing with difficult situations (ฉันเชื่อว่าฉันสามารถเรียนรู้ไปในทิศทางที่ดีขึ้นได้ เมื่อเผชิญกับสถานการณ์ที่ยากลำบาก)	5-point Likert scale: 1–2-3–4-5	3.6 (0.9); 4 (1–5)	2.1	16.1	− 0.44	3.00	0.87 (0.86–0.89)	0.76
Item 4: I actively look for ways to replace the losses I encounter in life (ฉันพยายามหาวิธีที่จะทดแทนความสูญเสียที่ฉันได้ประสบในชีวิต)	5-point Likert scale: 1–2-3–4-5	3.4 (1.0); 3 (1–5)	3.8	13.4	− 0.26	2.67	0.62 (0.59–0.64)	0.38
Overall	Possible range: 4 to 20	…	…	…	…	…	…	0.87
One-Dimensional Model Fit: Acceptable/Good (CFI, 0.991; TLI, 0.974; RMSEA, 0.085, SRMA, 0.019)^‡^								

^†^Based on standardized confirmatory factor analysis.

^‡^Threshold for acceptable fit: CFI, > 0.9; TLI, > 0.9; RMSEA, < 0.1; SRMR, < 0.1.

*BRCS* Brief Resilient Coping Scale, *CFI* comparative-fit index, *RMSEA* root mean square error of approximation, *SD* standard deviation, *SRMR* standardized root mean squared residual, *TLI* Tucker-Lewis index.

For the confirmatory factor analysis, satisfactory information criteria indices were found, the CFI (> 0.900), TLI (> 0.900), RMSEA (< 0.100), and SRMR (< 0.100) for a single latent component in the overall population and both sub-population cohorts, which indicates an acceptable model fit (Appendix, Table S2). Concerning the hierarchy of measurement invariance, no statistically significant differences were observed based on group differences (sample population, sexual identity, and religion), with all *P*-values > 0.05 at each level of the hierarchy (Appendix, Table S3). Standardized factor loadings for individual items based on the confirmatory factor analysis ranged from 0.62 to 0.87. The Thai-BRCS version, along with the original-BRCS version with four items and modeling indices is described in Table 2.

Moreover, a nonparametric item response theory analysis also reaffirmed the one-factor structure of the Thai-BRCS version according to unidimensionality, local independence, and monotonicity (Appendix, Table S4). For the scalability, all items for the whole sample and subpopulation cohort (general population and college students) had the *H*^*s*^ coefficients over 0.4, which indicate the strong scalability properties (Appendix, Table S4). Item characteristic curves for each item of the Thai-BRCS version are provided in Appendix, Fig. S1. The item categories illustrated that each item was informative for the total score, in that there was some point along the trait continuum regarding the same relationship. However, an inspection of individual items suggested that item 4 of the Thai-BRCS version showed a marginal uniform DIF regarding sexual identity (*P* = 0.019) and religion (*P* = 0.017) (Appendix, Table S5).

### Convergent and discriminant validity

Based on bivariate analysis, the Thai-BRCS version was statistically significantly correlated with the set of anchor-based measurement tools for convergent testing (*P* < 0.001 for all, Table 3). Overall, the Thai-BRCS version was slightly to moderately negatively correlated with PHQ-9 (correlation, − 0.28; 95% CI, − 0.31 to − 0.25), GAD-7 (correlation, − 0.36; 95% CI, − 0.38 to − 0.33), and PSS-10 (correlation, − 0.36; 95% CI, − 0.38 to − 0.33). Meanwhile, the Thai—BRCS version was moderately positively correlated with the WHO-5 (correlation, 0.43; 95% CI, 0.40 to 0.45) and MSPSS-12 (correlation, 0.38; 95% CI, 0.35 to 0.41). Furthermore, further analysis using multiple linear regression also revealed the correlation in terms of linearity of the Thai-BRCS version and the set of anchor-based measurement tools (*P* < 0.001 for all, Appendix, Table S6).

**Table 3:** Psychometric properties of the Thai-BRCS version questionnaire.

Psychometric properties	Overall (n = 4004)		General population (n = 2415)		College student (n = 1589)	
	Correlation (95% CI)^†^	*P* value	Correlation (95% CI)^†^	*P* value	Correlation (95% CI)^†^	*P* value
**Validity**						
Construct validity	A conceptual factorial structure was verified via exploratory and confirmatory factor analysis, and non-parametric item response theory					
Convergent validity						
With depression—PHQ-9	− 0.28 (− 0.31 to − 0.25)	< 0.001	− 0.28 (− 0.32 to − 0.24)	< 0.001	− 0.29 (− 0.34 to − 0.24)	< 0.001
With anxiety—GAD-7	− 0.28 (− 0.31 to − 0.25)	< 0.001	− 0.29 (− 0.33 to − 0.26)	< 0.001	− 0.26 (− 0.30 to − 0.21)	< 0.001
With perceived stress—PSS-10	− 0.36 (− 0.38 to − 0.33)	< 0.001	− 0.37 (− 0.40 to − 0.33)	< 0.001	− 0.34 (− 0.38 to − 0.29)	< 0.001
With well-being index—WHO-5	0.43 (0.40 to 0.45)	< 0.001	0.45 (0.42 to 0.49)	< 0.001	0.38 (0.34 to 0.43)	< 0.001
With perceived social support—MSPSS-12	0.38 (0.35 to 0.41)	< 0.001	0.41 (0.38 to 0.45)	< 0.001	0.32 (0.27 to 0.36)	< 0.001
Discriminant validity	Satisfactory with a known group comparison between the high resilient copers group and the medium and low resilient copers (details are listed in Table 4)					
**Reliability**						
Internal consistency: Cronbach’s α coefficient	0.84 (0.83 to 0.85)	< 0.001	0.86 (0.85 to 0.87)	< 0.001	0.80 (0.77 to 0.82)	< 0.001
Internal consistency: McDonald’s ω coefficient	0.85 (0.84 to 0.86)	< 0.001	0.87 (0.85 to 0.88)	< 0.001	0.80 (0.78 to 0.82)	< 0.001
Reproducibility: intraclass correlation	Based on the sub-cohort (n = 409): 0.91 (0.87 to 0.95), *P* value < 0.001					

^†^Pearson or Spearman rho correlation test.

*BRCS* Brief Resilient Coping Scale—4-items, *CI* confidence interval, *GAD-7* Generalized Anxiety Disorder—7-items, *MSPSS-12* Multidimensional Scale of Perceived Social Support—12-items, *PHQ-9* Patient Health Questionnaire—9-items, *PSS-10* Perceived Stress Scale—10-items, *SD* standard deviation, *WHO-5* World Health Organization Five Well-Being Index—5-items.

For discriminant validity, we used a known group of highly resilient copers as a reference. Multivariable logistic regression demonstrated that individuals with moderate psychological resilience, particularly, those with low resilience, had a positive association with a higher risk of adverse mental health outcomes, including depressive symptoms, anxiety, perceived stress, low well-being, and low perceived social support (Table 4). Moreover, the AuROC statistic also supported the discrimination performance of the Thai-BRCS version to distinguish individuals with and without adverse mental health outcomes; the AuROC ranged from 0.61 to 0.71 (Table 4).

**Table 4:** Discriminant validity of the Thai-BRCS version questionnaire.

Discriminant validity	Resilient coping^†^				AuROC (95% CI)
	Medium resilient copers: Thai-BRCS 14–16 points		Low resilient copers: Thai-BRCS 4–13 points		
	Adjusted OR (95% CI)^‡^	*P* value	Adjusted OR (95% CI)^‡^	*P* value	
**Depression: PHQ-9 ≥ 10 points**					
Overall (n = 4004)	1.28 (1.02–1.60)	0.032	2.62 (2.11–3.25)	< 0.001	0.62 (0.60–0.63)
General population (n = 2415)	1.41 (1.04–1.92)	0.025	2.78 (2.08–3.72)	< 0.001	0.62 (0.60–0.64)
College student (n = 1589)	1.17 (0.83–1.65)	0.373	2.36 (1.68–3.30)	< 0.001	0.61 (0.58–0.64)
**Anxiety: GAD-7 ≥ 8 points**					
Overall (n = 4004)	1.55 (1.18–2.04)	0.002	2.88 (2.22–3.74)	< 0.001	0.62 (0.60–0.63)
General population (n = 2415)	1.73 (1.20–2.49)	0.003	2.91 (2.05–4.14)	< 0.001	0.61 (0.58–0.64)
College student (n = 1589)	1.41 (0.93–2.13)	0.107	2.82 (1.90–4.20)	< 0.001	0.62 (0.59–0.65)
**Perceived stress: PSS-10 ≥ 14 points**					
Overall (n = 4004)	1.31 (1.08–1.58)	0.006	5.45 (4.38–6.78)	< 0.001	0.68 (0.67–0.70)
General population (n = 2415)	1.38 (1.08–1.76)	0.009	7.42 (5.58–9.88)	< 0.001	0.71 (0.69–0.73)
College student (n = 1589)	1.22 (0.88–1.70)	0.234	3.19 (2.23–4.58)	< 0.001	0.63 (0.61–0.66)
**Low well-being index: WHO-5 < 50 points**					
Overall (n = 4004)	1.81 (1.46–2.25)	< 0.001	5.26 (4.24–6.52)	< 0.001	0.67 (0.65–0.68)
General population (n = 2415)	1.96 (1.46–2.64)	< 0.001	6.78 (5.09–9.03)	< 0.001	0.69 (0.67–0.71)
College student (n = 1589)	1.69 (1.21–2.37)	0.002	3.69 (2.65–5.16)	< 0.001	0.64 (0.61–0.66)
**Low perceived social support: MSPSS-12 < 36 points**					
Overall (n = 4004)	0.97 (0.58–1.64)	0.923	2.85 (1.79–4.55)	< 0.001	0.64 (0.60–0.67)
General population (n = 2415)	1.04 (0.56–1.94)	0.894	2.93 (1.68–5.12)	< 0.001	0.63 (0.59–0.67)
College student (n = 1589)	0.88 (0.33–2.35)	0.793	2.72 (1.12–6.57)	0.026	0.64 (0.58–0.70)

^†^Reference group: high resilient copers (Thai-BRCS 17–20 points).

^‡^Adjusted for age, sexual identity, marital status, religion, living status, household family member, history of mental illness, quarantine status, and willingness to quarantine.

*AuROC* area under the receiver operating characteristic, *BRCS* Brief Resilient Coping Scale—4-items, *CI* confidence interval, *GAD-7* Generalized Anxiety Disorder—7-items, *MSPSS-12* Multidimensional Scale of Perceived Social Support—12-items, *PHQ-9* Patient Health Questionnaire—9-items, *PSS-10* Perceived Stress Scale—10-items, *WHO-5* World Health Organization Five Well-Being Index—5-items.

### Scale reliability

Regarding the overall population, the Thai-BRCS version revealed an excellent internal consistency in both Cronbach’s α (0.84; 95% CI, 0.83 to 0.85) and McDonald’s ω coefficient (0.85; 95% CI, 0.84 to 0.86). By separately subpopulation, the Cronbach’s α and McDonald’s ω coefficient were 0.86 (95% CI, 0.85 to 0.87) and 0.87 (95% CI, 0.85 to 0.88) for the general population; and (95% CI, 0.77 to 0.82) and 0.80 (95% CI, 0.78 to 0.82) for college students (Table 3). The item correlation between the Thai-BRCS for the single latent component, corrected item-total correlations, and Cronbach’s α and McDonald’s ω coefficient, if-item-deleted, is provided in the Appendix, Tables S7 and S8.

For scale stability, a sub-cohort of 409 participants who completed the Thai-BRCS for the second entry and available data were analyzed. The intraclass correlation coefficient test–retest reliability for the total score of the Thai-BRCS version was 0.91 (95% CI, 0.87 to 0.95), which denotes the almost perfect scale stability (Table 3).

## Discussion

To help healthcare professionals, public health officials, and public society by quantifying, identifying people at risk, and targeting optimal strategies to the coping population during unprecedented situations, a valid, reliable, and practical tool for measuring psychological resilience is needed. This study provides a suitable scale and addresses the first evidence, to our knowledge, of the transcultural adaptation and validity of the simple four-item Thai-BRCS version, a self-reported measure of resilient coping in the Thai population (general population and college students) during the COVID-19 pandemic.

The Thai-BRCS version was created and verified using a multidimensional approach, including exploratory and confirmatory factor analysis and nonparametric item response theory analysis. These findings reaffirmed the construct validity of the scale factor structure with a one-factor component assumption and suggested acceptable convergent and discriminant validity. Meanwhile, the summary score of the Thai-BRCS version convergent moderately positively with the well-being index (WHO-5 scale) and perceived social support (MSPSS-12 scale), the Thai-BRCS version was slightly to moderately negatively correlated with depression (PHQ-9 scale), anxiety (GAD-7 scale), and perceived stress (PSS-10 scale). All significant correlations between the Thai-BRCS version and set of anchor-based mental health and psychosocial measurement tools also reflected the conceptualization of the scale.

Although the measurement invariance established observed and latent estimated in all multiple groups analyzed (sample population, sexual identity, and religion). However, according to the DIF analyses, females and individuals with self-reported irreligion had a slightly higher ability to overcome substantial stressors; “item-4, I actively look for ways to replace the losses I encounter in life” compared with males/others and the person's self-identification as religion (Buddhist/Christian/Muslim/Others), respectively. Recently, evidence revealed disparities in COVID-19-related public stigma, especially among males and Buddhists in Thailand^43^. In this circumstance, we hypothesized that some hidden residual traits and characteristics may mediate and contribute to the ability and capacity to coping the negative consequence in both general situations and during the pandemic. Moreover, psychological responses and coping to major disasters or public infectious events may also depend on external individual factors such as inequality and poverty impacts, government policy responses, and preparedness of countries in terms of healthcare expenditure and economic responses^1^. To enhance the interpretability of the psychological resilience at a global level regarding the effect of cultural background, an international study is needed to account for the DIF across countries.

The Thai-BRCS version also revealed good scale reliability in terms of internal reliability and scale stability. In addition, no substantial differences were observed in terms of the Cronbach’s α and McDonald’s ω coefficient after removing any items from the Thai-BRCS version, demonstrating the robustness of the internal reliability and interrelation of the scale. For scale feasibility and practicability, this scale is easy to use by the general public and useful to implement in the public health survey, as it can be completed in less than 5 min. As a result, this scale can also be a great advantage when used multiple times longitudinally.

Collectively, one major finding of our study is the evidence of measurement across the Thai population, including the general population and college students, comparable to previous studies using the original English and non-English-BRCS version^13–18^. In previous studies, the internal consistency reliability ranged from 0.68 to 0.87. This study found that the Thai-BRCS version had a Cronbach’s α and McDonald’s ω coefficient of 0.84 (95% CI, 0.83 to 0.85) and (0.85; 95% CI, 0.84 to 0.86), respectively. However, college students appear to have an internal consistency lower than that of the general population (both Cronbach’s α and McDonald’s ω coefficient). With respect to the subpopulation cohort, the general population also seems to have a better discriminative performance than college students in all aspects of the anchor-based measurement of mental health and psychosocial tools. We postulated that middle or older adulthood may experience countering the negative effects of stressors and may have psychological resilience perception, emotional regulation, and coping style differences from young adulthood. However, further studies are warranted to confirm these findings.

Furthermore, our findings also highlight and replicate the associations between the degree of psychological resilience and the risk of adverse mental outcomes. Compared with the high resilience copers group, individuals with low psychological resilience had a significant association with depression, anxiety, perceived stress, low well-being index, and low perceived social support in the general population and college students (Table 4). These results support the concept that resilience is the capacity to adapt and overcome significant stressors^5,6^.

### Strengths and limitations

This study was conducted with a rigorous and comprehensive method, and we translated the original English-BRCS version into the Thai-BRCS version using the standard approach. Based on the public’s values and perspectives, a sophisticated quantitative approach was used to validate and assess the psychometric properties of the Thai-BRCS version. Furthermore, this scale was verified and reaffirmed the conceptual concept of psychological resilience during the COVID-19 pandemic, reflecting Thai cultural norms in facing and responding to major public health events.

However, this study has some limitations. First, to adhere to the physical distancing strategy during the first lockdown in Thailand, only participants who could access the Internet were included in this study. Therefore, selection bias and respondent bias should be noted. Second, although the psychometric properties of the Thai-BRCS version are fulfilled by transcultural adaptation in terms of validity and reliability in both the general population and college students, which can adopt this scale for use in a broad population. However, our study may not represent the elderly population, as the included participants were based on young adulthood to middle adulthood. To overcome this issue, future studies with a more diverse population, particularly those of advanced age, as well as other specific groups (i.e., minorities and vulnerable groups or healthcare workers), are recommended to expand the generalizability and utility of this scale. Lastly, this study lacks information on public response to change and long-term scale effects in terms of minimum clinically important differences. To fill this gap and help target specific interventions regarding psychological resilience, responsiveness validity, and long-term tracking of public resilient coping through longitudinal evaluation are warranted.

### Practical implications of the Thai-BRCS

The Thai-BRCS scale is also practical and can now be used in a public health survey to support policy decisions. This scale can be incorporated into research and implemented as a tool to identify people at risk for adverse mental health outcomes during the COVID-19 pandemic. Additionally, the Thai-BRCS can be useful to evaluate the effectiveness or responsiveness of interventions.

## Conclusions

In summary, the Thai-BRCS version is suitable for measuring psychological resilience in the Thai population and fulfills cross-cultural adaptation with satisfactory psychometric properties for validity and reliability. The Thai-BRCS scale is also practical and can now be used in a public health survey to support policy decisions. This scale can be incorporated into research and implemented as a tool to identify people at risk for adverse mental health outcomes during the COVID-19 pandemic. However, to improve generalizability and long-term utility, more studies on the validity of public responsiveness are warranted to detect the performance of the scale, including its sensitivity to change and minimum clinically significant.

## Supplementary Information

Supplementary Information.

## Data Availability

Data will be shared upon reasonable request and with permission according to the Health Outcomes and Mental Health Care Evaluation Survey Research Group (HOME-Survey) data release policy.

## Code availability

The analytic Stata code for the primary analysis is available at https://zenodo.org/record/6969080#.Yu4QjRxBw2w (10.5281/zenodo.6969080).
