# Supplementary material for: Transcultural adaptation and psychometric validation of the Thai-Brief Resilient Coping Scale: a cross-sectional study during the coronavirus disease 2019 pandemic in Thailand
Source: Sci Rep. 2022 Dec 13;12:21521. doi: 10.1038/s41598-022-26063-8 (PMC9745707; doi:10.1038/s41598-022-26063-8)
Supplement: Supplementary file 1 — Supplementary Information. [file 41598_2022_26063_MOESM1_ESM.pdf]

Supplementary Appendix

Transcultural Adaptation and Psychometric Validation of the Thai-Brief Resilient Coping Scale: A Cross-Sectional Study During the Coronavirus Disease 2019 Pandemic in Thailand

Surapon Nochaiwong*, Chidchanok Ruengorn, Ratanaporn Awiphan, Chabaphai Phosuya, Yongyuth Ruanta, Penkarn Kanjanarat, Nahathai Wongpakaran, Tinakon Wongpakaran, Kednapa Thavorn

***Correspondence and requests for materials:**

Surapon Nochaiwong, PharmD, Department of Pharmaceutical Care, Faculty of Pharmacy, Chiang Mai University, Chiang Mai 50200, Thailand, Phone: 66899973365, Fax: 6653222741, Email: surapon.nochaiwong@gmail.com

Supplementary Online Content

**eMethods**	Methods for Measurement Invariance Approach	S3
**Table S1**	Exploratory Factor Analysis of the Thai-BRCS Version	S4
**Table S2**	Confirmatory Factor Analysis of the Thai-BRCS Version	S5
**Table S3**	Hierarchy of Measurement Invariance for the Thai-BRCS Version	S6
**Table S4**	Nonparametric Item Response Theory Analysis of the Thai-BRCS Version	S7
**Table S5**	Differential Item Functioning for the Thai-BRCS Version	S8
**Table S6**	Multiple Linear Regression Analyses Examining Association of the Thai-BRCS Version with Mental Health and Psychosocial Problems	S9
**Table S7**	Item Correlation Among the Thai-BRCS Version	S10
**Table S8**	Internal reliability of the Thai-BRCS Version	S11
**Figure S1**	Item Characteristics Curves of the Thai-BRCS Version	S12

eMethods Methods for Measurement Invariance Approach

Regarding the suggested criteria for established model measurement invariance, a hierarchical approach was performed across different groups, including sample population (general population or college students), sexual identity (male, female, or others), and religion (irreligion, Buddhist, or Christian/Muslim/others). The model parameters were subjected to sequential stringent restrictions, and changes in the model fit indices were also tested. To verify measurement invariance, the configured invariance model had to be established and compared to the fit indices for other invariance models consecutively based on an increasingly restrictive, stepwise manner.

The measurement models were tested by imposing equality constraints on parameters across different groups. First, the configural invariance model placed no pre-specified variable groups (i.e., sample population, sexual identity, and religion) equality constraints on model parameters. Second, the weak invariance (metric) model was then constructed and assessed. The weak invariance model employed equality constraints on model factor loadings across pre-specified variable groups (i.e., sample population, sexual identity, and religion), allowing the scale factors to be compared across different groups. Third, scalar or strong invariance was tested; the intercepts were set to be invariant in addition to the factor loadings, In the case of scalar invariance holds, it was possible to compare the factor means across different groups. Forth, a strict invariance (residuals) model was developed and assessed to ensure that the error item variance was invariant across pre-specified variable groups. Of these, the rigorous invariance model was based on equality constraints on the factor loadings, item intercepts, and residual variances. Lastly, strict invariance plus factor means were performed to determine the factor mean differences between pre-specified variable groups. The structure invariance measured the amount of difference across pre-specified variable groups and had no relation to the measurement scale investigated.

References

1. Barbara, M. B. Structural Equation Modeling with Mplus: Basic Concepts, Applications, and Programming. (Routledge, 2011).
2. van de Schoot, R., Lugtig, P. & Hox, J. A checklist for testing measurement invariance. European Journal of Developmental Psychology. 9, 486-492 (2012).
3. Alan, C. A. Discovering Structural Equation Modeling Using Stata, Revised Edition. (Stata Press, 2013).
4. Jichuan, W. & Xiaoqian, W. Structural Equation Modeling: Applications Using Mplus. 2nd edn, (John Wiley & Sons, 2020).

Table S1 Exploratory Factor Analysis of the Thai-BRCS Version

**Exploratory Factor Analysis: PCA†: Item**	**Factor Loading: One Factor**	**Communality Value**
**Overall (n=2,002)**		
Q1	0.82	0.67
Q2	0.85	0.73
Q3	0.88	0.78
Q4	0.74	0.55
Total Percentage of the variance: 0.68		
**General population (n=1,207)**		
Q1	0.83	0.70
Q2	0.87	0.76
Q3	0.90	0.81
Q4	0.76	0.58
Total Percentage of the variance: 0.71		
**College student (n=795)**		
Q1	0.78	0.61
Q2	0.82	0.67
Q3	0.85	0.72
Q4	0.71	0.51
Total Percentage of the variance: 0.63		

†The extraction method based on the rotation method by orthogonal, varimax rotation.

Abbreviations: BRCS, Brief Resilient Coping Scale; PCA, principle component analysis.

Table S2 Confirmatory Factor Analysis of the Thai-BRCS Version

1-Dimensional Model	Threshold for Acceptable Fit					Model fit
	CFI (>0.9)	TLI (>0.9)	RMSEA (<0.1)	SRMR (<0.1)	R-Squared (>0.30)	
Overall (n=2,002)	0.991	0.974	0.085	0.019	Q1 = 0.51<br>Q2 = 0.67<br>Q3 = 0.76<br>Q4 = 0.38	Acceptable/Good
General population (n=1,208)	0.996	0.989	0.060	0.013	Q1 = 0.56<br>Q2 = 0.71<br>Q2 = 0.80<br>Q3 = 0.42	Acceptable/Good
College student (n=794)	0.979	0.938	0.087	0.030	Q1 = 0.43<br>Q2 = 0.59<br>Q2 = 0.70<br>Q3 = 0.32	Acceptable/Good

Abbreviations: BRCS, Brief Resilient Coping Scale; CFI, comparative-fit index; RMSEA, root mean square error of approximation; SRMR, standardized root mean squared residual; TLI, Tucker-Lewis index.

Table S3 Hierarchy of Measurement Invariance for the Thai-BRCS Version

**Model**	**Sample Population**					
	χ^2	**Degree of Freedom**	**Ref. Model**	**Delta χ^2**	**Delta Degree of Freedom**	***P*-Value**
Configural invariance	2157.15	6	-	-	-	-
Metric invariance	2164.01	9	1	6.86	3	0.076
Scalar invariance	2165.90	12	2	1.89	3	0.596
Strict invariance	2172.60	15	3	6.70	3	0.082
Strict invariance + factor means	2172.63	16	4	0.03	1	0.862
**Model**	**Sexual Identity**					
	χ^2	**Degree of Freedom**	**Ref. Model**	**Delta χ^2**	**Delta Degree of Freedom**	***P*-Value**
Configural invariance	2134.92	9	-	-	-	-
Metric invariance	2144.33	15	1	9.41	6	0.152
Scalar invariance	2152.69	21	2	8.36	6	0.212
Strict invariance	2158.70	27	3	6.01	6	0.422
Strict invariance + factor means	2159.51	29	4	0.81	2	0.667
**Model**	**Religion**					
	χ^2	**Degree of Freedom**	**Ref. Model**	**Delta χ^2**	**Delta Degree of Freedom**	***P*-Value**
Configural invariance	2109.11	9	-	-	-	-
Metric invariance	2119.15	15	1	10.04	6	0.123
Scalar invariance	2127.58	21	2	8.43	6	0.208
Strict invariance	2138.92	27	3	11.34	6	0.078
Strict invariance + factor means	2140.31	29	4	1.39	2	0.499

Abbreviations: BRCS, Brief Resilient Coping Scale.

Table S4 Nonparametric Item Response Theory Analysis of the Thai-BRCS Version

**Item**	**Loevinger's H Coefficients (H^s)†**	**Z-statistics**	***P*-Value**	**Monotonicity Assumption (Criterion <80)**
**Overall (n=4,004)**				
Q1	0.61	61.44	<0.001	-15
Q2	0.63	64.71	<0.001	-17
Q3	0.68	68.07	<0.001	-19
Q4	0.54	54.94	<0.001	-12
**General population (n=2,415)**				
Q1	0.64	51.11	<0.001	-17
Q2	0.67	54.13	<0.001	-19
Q3	0.72	56.52	<0.001	-21
Q4	0.57	45.91	<0.001	-14
**College student (n=1,589)**				
Q1	0.54	33.88	<0.001	-12
Q2	0.55	35.28	<0.001	-13
Q3	0.61	37.71	<0.001	-15
Q4	0.48	30.24	<0.001	-9

†Loevinger's H Coefficients indicates that, if H^s <0.3, the scale has poor scalability properties; 0.3 ≤ H^s <0.4, the scale is weak; 0.4 ≤ H^s <0.5, the scale is medium; and H^s ≥0.5, the scale is strong.

Abbreviations: BRCS, Brief Resilient Coping Scale.

Table S5 Differential Item Functioning for the Thai-BRCS Version

Item	Differential Item Functioning								
	Sample Population (General Population)†			Sex (Female)‡			Religion (Irreligion)§		
	χ^2	OR (95% CI)	*P*-Value	χ^2	OR (95% CI)	*P*-Value	χ^2	OR (95% CI)	*P*-Value
Q1	0.01	1.01 (0.75 – 1.37)	0.998	2.25	0.78 (0.57 – 1.06)	0.133	3.52	0.62 (0.38 – 1.01)	0.061
Q2	1.96	1.26 (0.93 – 1.71)	0.162	0.02	1.03 (0.76 – 1.42)	0.896	0.01	0.98 (0.60 – 1.60)	0.959
Q3	0.01	1.03 (0.76 – 1.40)	0.911	1.46	0.81 (0.59 – 1.12)	0.227	0.27	0.85 (0.52 – 1.39)	0.602
Q4	2.30	0.80 (0.60 – 1.05)	0.130	5.49	1.43 (1.07 – 1.92)	0.019	5.67	1.73 (1.12 – 2.66)	0.017

†Compared with subpopulation—college student.

‡Compared with male/others.

§Compared with religion (Buddhist/Christian/Muslim/Others).

Abbreviations: BRCS, Brief Resilient Coping Scale; CI, confidence interval; OR, odds ratio.

Table S6 Multiple Linear Regression Analyses Examining Association of the Thai-BRCS Version with Mental Health and Psychosocial Problems

Mental Health and Psychosocial Metrics	Unadjusted Model			Adjusted Model†		
	Coefficient β (95% CI)	*P*-Value	R^2	Coefficient β (95% CI)	*P*-Value	R^2
**Overall (n=4,004)**						
Depression—PHQ-9	-0.47 (-0.53 to -0.41)	<0.001	0.06	-0.38 (-0.43 to -0.32)	<0.001	0.24
Anxiety—GAD-7	-0.35 (-0.40 to -0.30)	<0.001	0.05	-0.29 (-0.33 to -0.24)	<0.001	0.17
Perceived stress—PSS-10	-0.74 (-0.80 to -0.68)	<0.001	0.11	-0.66 (-0.72 to -0.60)	<0.001	0.24
Well-being index—WHO-5	2.91 (2.71 to 3.11)	<0.001	0.17	2.71 (2.52 to 2.90)	<0.001	0.26
Perceived social support—MSPSS-12	1.75 (1.62 to 1.88)	<0.001	0.15	1.68 (1.55 to 1.80)	<0.001	0.20
**General population (n=2,415)**						
Depression—PHQ-9	-0.43 (0.50 to -0.35)	<0.001	0.05	-0.33 (-0.40 to 0.27)	<0.001	0.23
Anxiety—GAD-7	-0.32 (-0.38 to -0.27)	<0.001	0.05	-0.26 (-0.31 to -0.20)	<0.001	0.17
Perceived stress—PSS-10	-0.71 (-0.79 to -0.63)	<0.001	0.12	-0.64 (-0.72 to -0.57)	<0.001	0.24
Well-being index—WHO-5	2.94 (2.70 to 3.18)	<0.001	0.19	2.80 (2.56 to 3.04)	<0.001	0.27
Perceived social support—MSPSS-12	1.83 (1.67 to 1.99)	<0.001	0.17	1.76 (1.60 to 1.93)	<0.001	0.21
**College student (n=1,589)**						
Depression—PHQ-9	-0.57 (-0.67 to -0.46)	<0.001	0.07	-0.44 (-0.54 to -0.34)	<0.001	0.21
Anxiety—GAD-7	-0.41 (-0.48 to -0.32)	<0.001	0.06	-0.32 (-0.40 to -0.24)	<0.001	0.15
Perceived stress—PSS-10	-0.80 (-0.91 to -0.69)	<0.001	0.11	-0.67 (-0.78 to -0.56)	<0.001	0.22
Well-being index—WHO-5	2.84 (2.51 to 3.18)	<0.001	0.14	2.51 (2.18 to 2.84)	<0.001	0.22
Perceived social support—MSPSS-12	1.59 (1.38 to 1.81)	<0.001	0.12	1.45 (1.24 to 1.66)	<0.001	0.17

†Adjusted for age, sexual identity, marital status, religion, living status, household family member, history of mental illness, quarantine status, and willingness to quarantine.

Abbreviations: BRCS, Brief Resilient Coping Scale—4-items; CI, confidence interval; GAD-7, Generalized Anxiety Disorder—7-items; MSPSS-12, Multidimensional Scale of Perceived Social Support—12-items; PHQ-9, Patient Health Questionnaire—9-items; PSS-10, Perceived Stress Scale—10-items; WHO-5, World Health Organization Five Well-Being Index—5-items.

Table S7 Item Correlation Among the Thai-BRCS Version

**Overall (n=4,004)**	**Q1**	**Q2**	**Q3**	**Q4**
**Q1**	1.000			
**Q2**	0.58 (*P*<0.001)	1.000		
**Q3**	0.61 (*P*<0.001)	0.73 (*P*<0.001)	1.000	
**Q4**	0.49 (*P*<0.001)	0.48 (*P*<0.001)	0.54 (*P*<0.001)	1.000
**General population (n=2,415)**	**Q1**	**Q2**	**Q3**	**Q4**
**Q1**	1.000			
**Q2**	0.63 (*P*<0.001)	1.000		
**Q3**	0.66 (*P*<0.001)	0.76 (*P*<0.001)	1.000	
**Q4**	0.52 (*P*<0.001)	0.52 (*P*<0.001)	0.58 (*P*<0.001)	1.000
**College student (n=1,589)**	**Q1**	**Q2**	**Q3**	**Q4**
**Q1**	1.000			
**Q2**	0.50 (*P*<0.001)	1.000		
**Q3**	0.53 (*P*<0.001)	0.66 (*P*<0.001)	1.000	
**Q4**	0.45 (*P*<0.001)	0.39 (*P*<0.001)	0.47 (*P*<0.001)	1.000

Abbreviation: BRCS, Brief Resilient Coping Scale.

Table S8 Internal reliability of the Thai-BRCS Version

Items	Corrected Item-Total Correlation	Cronbach's Alpha Coefficient if Item Deleted	McDonald's Omega Coefficient if Item Deleted
**Overall (n=4,004)**			
**Q1**	0.81	0.80	0.82
**Q2**	0.84	0.78	0.79
**Q3**	0.87	0.76	0.77
**Q4**	0.77	0.84	0.85
**General population (n=2,415)**			
**Q1**	0.83	0.83	0.84
**Q2**	0.86	0.81	0.81
**Q3**	0.89	0.79	0.79
**Q4**	0.78	0.87	0.87
**College students (n=1,589)**			
**Q1**	0.78	0.76	0.77
**Q2**	0.81	0.74	0.74
**Q3**	0.84	0.71	0.71
**Q4**	0.73	0.79	0.80

Abbreviation: BRCS, Brief Resilient Coping Scale.

Figure S1 Item Characteristics Curves of the Thai-BRCS Version

Q1

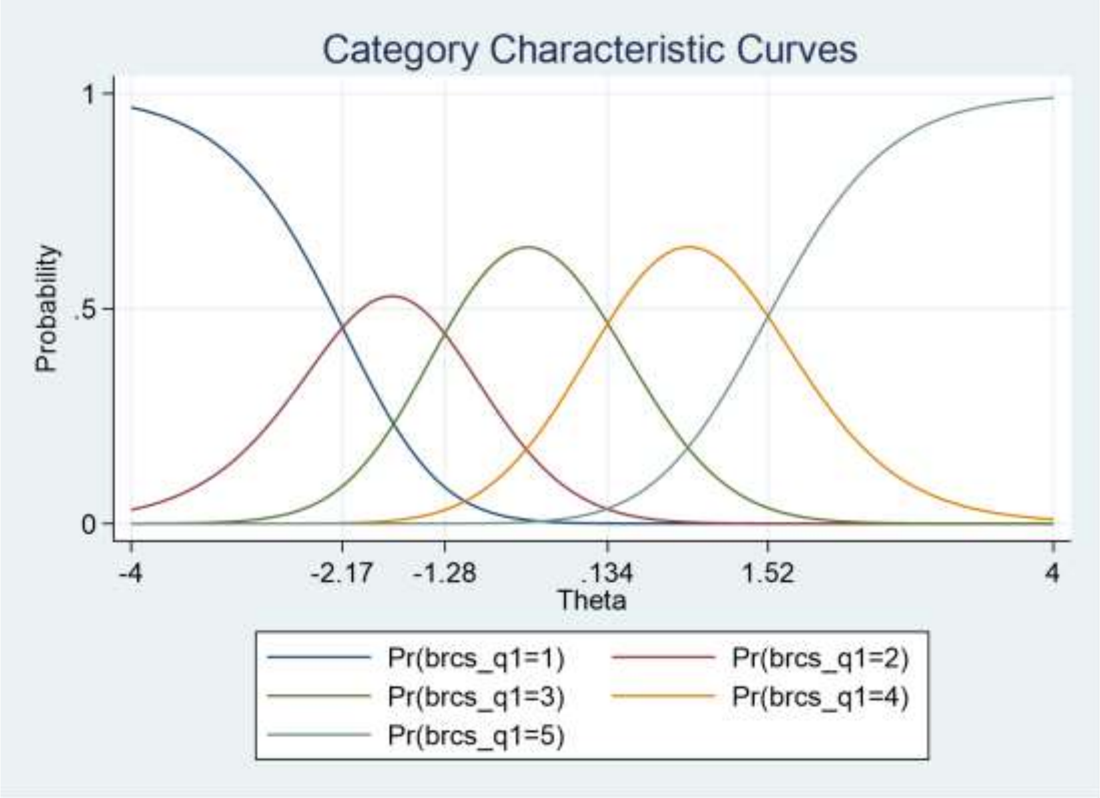

Q2

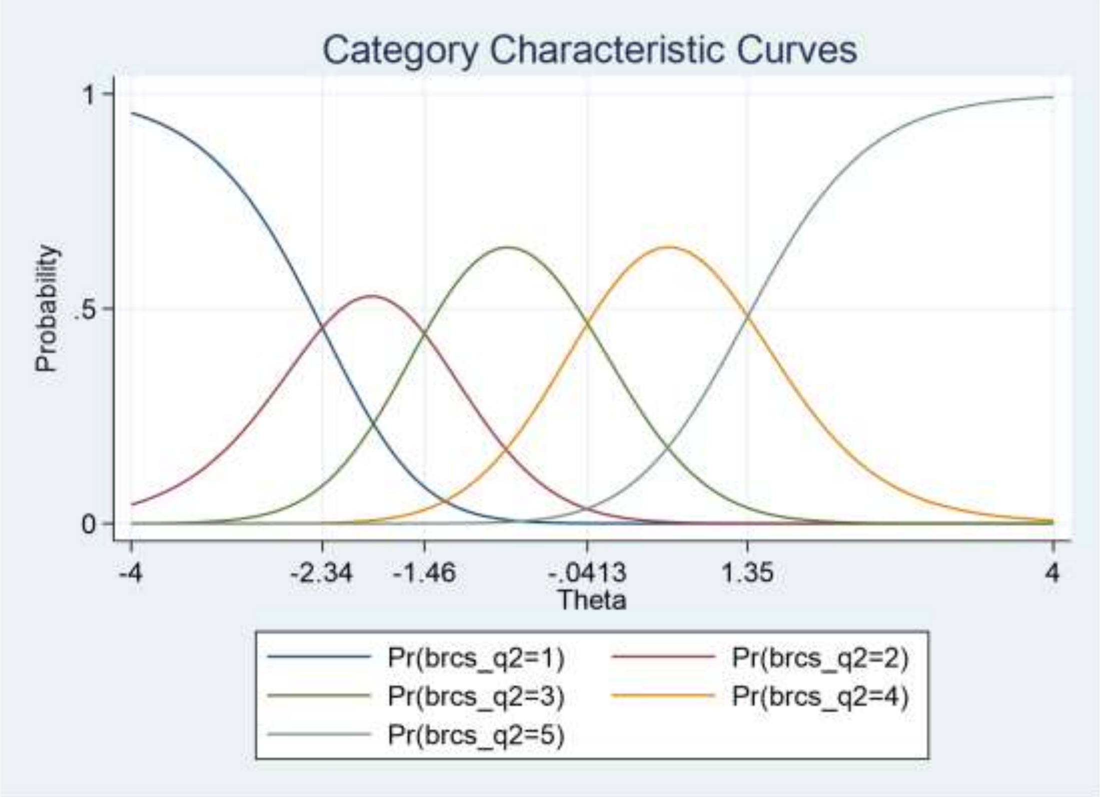

Abbreviation: BRCS, Brief Resilient Coping Scale.

Figure S1 Item Characteristics Curves of the Thai-BRCS Version (Continued)

Q3

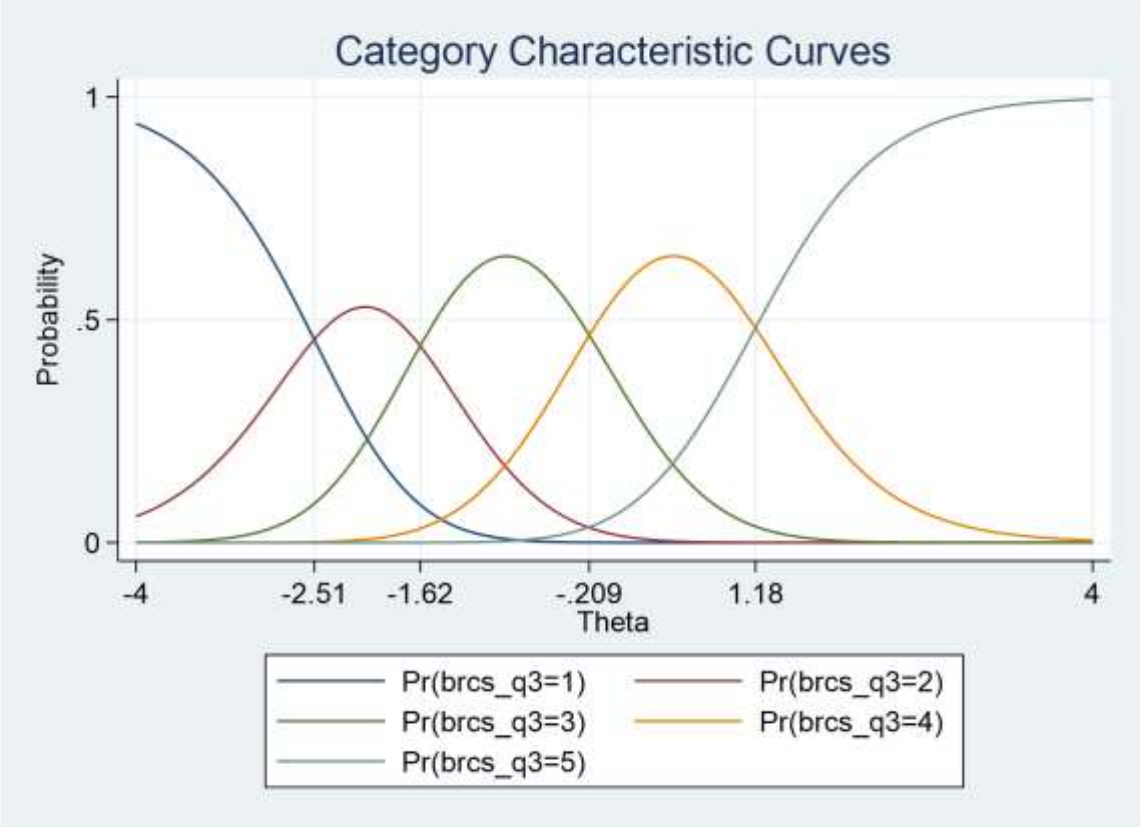

Q4

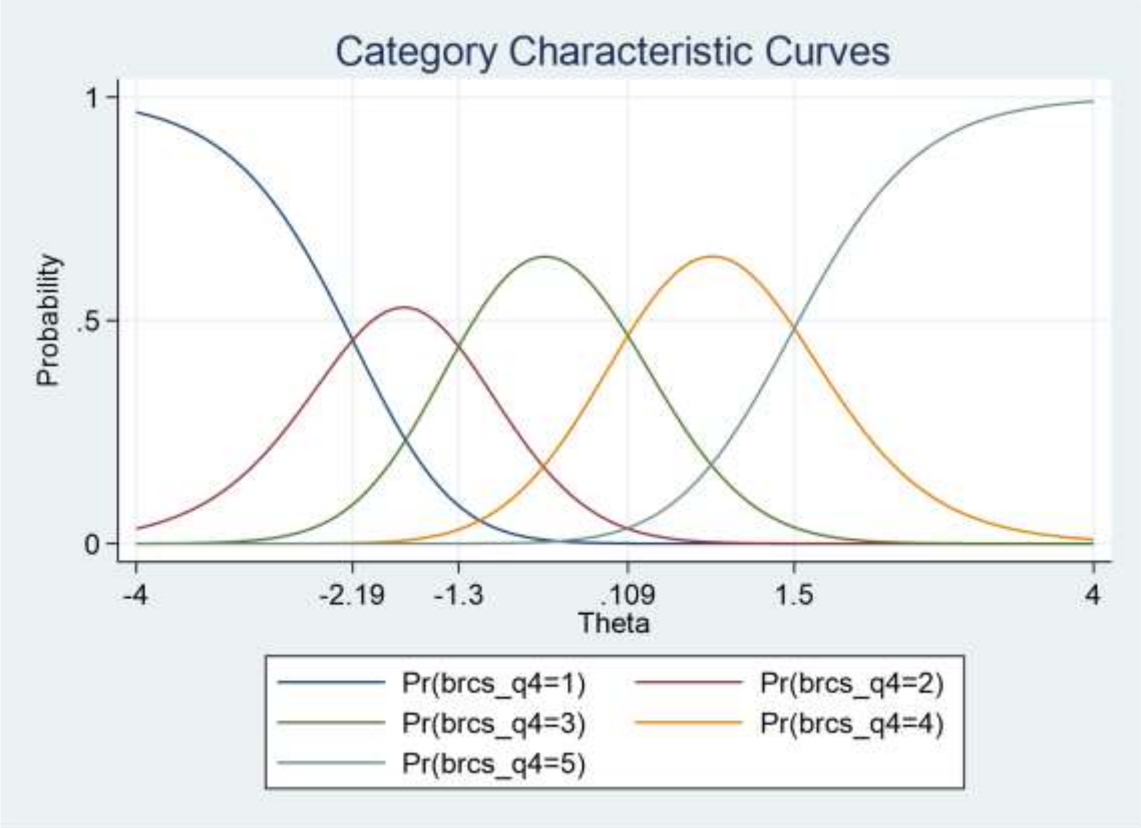

Abbreviation: BRCS, Brief Resilient Coping Scale.
